# The Potential Lubricating Mechanism of Alginate Acid and Carrageenan on the Inner Surface of Orthokeratology Lenses

**DOI:** 10.3390/polym18010004

**Published:** 2025-12-19

**Authors:** Chen-Ying Su, Lung-Kun Yeh, You-Cheng Chang, Pei-Ting Lu, Yung-Hsiang Chang, Kuo-Hsuan Hung, Chi-Chun Lai, Hsu-Wei Fang

**Affiliations:** 1 Department of Chemical Engineering and Biotechnology, National Taipei University of Technology, No. 1, Sec. 3, Zhongxiao E. Rd., Taipei 106, Taiwan; chenying.su@ntut.edu.tw (C.-Y.S.); youchengchang@gmail.com (Y.-C.C.); zoop7377@gmail.com (P.-T.L.); you77551@gmail.com (Y.-H.C.); 2 High-Value Biomaterials Research and Commercialization Center, National Taipei University of Technology, No. 1, Sec. 3, Zhongxiao E. Rd., Taipei 106, Taiwan; 3 Department of Ophthalmology, Chang Gung Memorial Hospital, Linkou, No. 5, Fuxing St., Taoyuan 333, Taiwan; yehlungkun@gmail.com (L.-K.Y.); scrapie@cgmh.org.tw (K.-H.H.); 4 College of Medicine, Chang Gung University, No. 259, Wenhua 1st Rd., Taoyuan 333, Taiwan; chichun.lai@gmail.com; 5 Department of Ophthalmology, Chang Gung Memorial Hospital, Keelung, No. 222, Maijin Rd., Keelung City 204, Taiwan; 6 Institute of Biomedical Engineering and Nanomedicine, National Health Research Institutes, No. 35, Keyan Road, Miaoli 350, Taiwan; 7 Institute of Oral Tissue Engineering and Biomaterials, National Yang Ming Chiao Tung University, Taipei 112, Taiwan

**Keywords:** orthokeratology lens, alginate acid, lambda-carrageenan, tribological property, protein adsorption and desorption, zeta potential, turbidity

## Abstract

When an orthokeratology (ortho-k) lens contacts the ocular surface, tear film components such as lipids and proteins rapidly adsorb onto the lens, which may increase friction and contribute to discomfort if not properly removed. Polysaccharides have been reported to reduce protein deposition and improve lubrication, prompting the investigation of alginate acid and lambda-carrageenan in modulating the tribological properties of ortho-k lenses. An in vitro tribological property analysis of ortho-k lenses and protein adsorption and desorption analyses were carried out to investigate the lubricating ability of alginate acid and carrageenan. Zeta potential and turbidity analyses were further conducted to examine potential interactions between polysaccharides and tear film proteins. Tear film proteins significantly increased the friction coefficient of the ortho-k lens, whereas the addition of alginate acid and carrageenan markedly reduced friction. Electrostatic interaction and polysaccharide–protein complex formation were identified as possible mechanisms underlying these effects. These results demonstrate that alginate acid and carrageenan can modify the tribological and interfacial behavior of ortho-k lenses in protein-rich environments, suggesting their potential application in reducing friction-related complications in ortho-k lens wearers.

## 1. Introduction

The global prevalence of myopia continues to rise and is expected to reach approximately half of the world’s population by 2025 [1]. For visual convenience and aesthetic reasons, many people rely on contact lenses. Although soft hydrogel or silicone hydrogel contact lenses remain the most common choices, the use of orthokeratology (ortho-k) lenses, originally derived from rigid gas-permeable (RGP) lenses, has become increasingly popular among schoolchildren [2]. Ortho-k lenses incorporate a reverse geometry design that temporarily reshape the anterior cornea during overnight wear, enabling clear daytime vision without the need for correction [3]. These ortho-k lenses are primarily composed of polymethylmethacrylate (PMMA), and the incorporation of silicone acrylate enhances both oxygen permeability and hydrophilicity; Nevertheless, the lens surface remains predominantly hydrophobic, which can contribute to ocular discomfort [4]. The hydrophobic regions interact with the nonpolar tails of tear film lipids, leaving their hydrophilic segments exposed and subsequently promoting protein adsorption [5]. Without effective cleaning, tear film proteins may progressively accumulate on the ortho-k lens surface [6,7]. It has been shown that tear film protein deposition can elevate the friction coefficient of hydrogel and silicone hydrogel contact lenses [8,9,10], and the same phenomenon was also observed for RGP lenses sliding in artificial tear solution containing tear film proteins such as lysozyme and albumin [11]. Since the in vitro friction coefficient of contact lenses is considered to be related to the degree of comfort in vivo [12], protein-induced increases in the friction of ortho-k lenses may partly explain discomfort by some ortho-k lens wearers. Therefore, reducing the friction coefficient of the ortho-k lens will be critical.

Extensive research has explored frictional behavior using in vitro tribological analyses for hydrogel and silicone hydrogel contact lenses. To simulate physiological interactions, researchers have used ex vivo human or mouse corneal tissues as opposing substrates [13,14]. In order to avoid biological variability, alternative counter surfaces such as a mucin-coated glass disk or a quartz glass disk have also been employed [8,15]. Recently, gelatin substrates, owing to their similarity to ocular tissue, have been used for evaluating the tribology of RGP lenses [11,16,17]. Unlike soft contact lens wearers, individuals using ortho-k lenses experience limited eyelid-lens or cornea-lens movement during sleep. However, when an individual enters the rapid eye movement stage of sleep, tribological movement takes place between the anterior cornea and the inner surface of the ortho-k lens. It will be more suitable if the tribological properties on the inner surface of the ortho-k lens is investigated.

Lowering the friction experienced at the surface of ortho-k lenses may be achieved either by removing protein deposits or by enhancing lubrication. Standard care instructions for ortho-k lenses involve soaking the lenses in a multipurpose care solution after removal each day, along with the use of hydrogen peroxide or enzymes on a weekly basis for more intensive cleaning [18]. While these products are formulated to disinfect, clean, and store lenses, lubrication is not their primary function [19]. Therefore, a daily care solution that can simultaneously clean and improve surface lubrication could help alleviate discomfort for ortho-k lens users. Alginate acid (AA) and lambda-carrageenan (CRG) are naturally derived polymers widely employed in various biomedical applications [20,21]. AA can form hydrogels in the presence of divalent ions such as calcium, and such hydrogels have been reported to exhibit favorable lubricating behavior [22,23]. Carrageenans exist in three major forms including iota-, kappa-, and lambda-carrageenan. Iota-carrageenan has been incorporated into microparticle sprays capable of providing effective lubrication [24]. Kappa-carrageenan, particularly in hydrogel form, has been shown to reduce friction between solid surfaces including glass and stainless steel under specific conditions [25,26]. Moreover, formulations combining kappa- and lambda-carrageenan have gained medical device approval as virginal gels due to their dual lubricating and antimicrobial characteristics [27]. Previous work in artificial joint lubrication has indicated that mixtures of AA and lambda-CRG can significantly decrease friction derived by human serum albumin [28]. Additional findings suggest that AA/CRG combinations can reduce protein adsorption on ortho-k lens surfaces when incorporated into artificial tear solution [29]. Despite these insights, whether AA and lambda-CRG can also provide lubrication directly on ortho-k lens surfaces remains unknown.

In the present study, an in vitro system to examine the tribological behavior of the inner surface of ortho-k lenses was established. The ortho-k lenses were tested by sliding them against a quartz pin in artificial tear solution, commercial care solution, or artificial tears supplemented with AA and CRG. The viscosity of AA + CRG care solution was measured, and adsorption/desorption assessments were performed using a quartz crystal microbalance to clarify their potential lubrication mechanisms. Finally, possible interactions between AA/CRG and tear film proteins including electrostatic effects and complex formation were explored through zeta potential measurements and turbidity analysis.

## 2. Materials and Methods

### 2.1. Orthokeratology Lens and Reagents

One semi-final product of orthokeratology (ortho-k) lenses was used for tribological testing, Boston ES (the generic USAN material name is Enflufocon A) was manufactured by Brighten Optix Co. (Taipei, Taiwan). The commercial care solution used in the present study was MeniCare Plus multipurpose solution for all rigid gas permeable lenses (Menicon, Nagoya, Japan), and the main ingredients were poloxamer and hypromellose. For tribological property analysis, adsorption and desorption analyses, and viscosity tests, alginate acid (AA, molecular weight is 120,000–190,000 g/mol, Sigma–Aldeich, St. Louis, MO, USA) and carrageenan (CRG, molecular weight is 560,500–580,500 g/mol, Sigma–Aldeich) were prepared as an ortho-k care solution. The AA + CRG care solution contained 0.45 g of AA and 0.45 g of lambda-CRG, resulting in a final concentration of 4.5 mg/mL AA and 4.5 mg/mL CRG in the care solution. Previous work has investigated the protein deposit removal ability of AA and CRG with the final concentration as 2.25 mg/mL or 4.5 mg/mL, and the results indicated that 4.5 mg/mL of AA + CRG can effectively remove adsorbed proteins from the ortho-k lens surface [29]. Thus, the care solution containing 4.5 mg/mL AA and 4.5 mg/mL CRG was analyzed for its lubricating property in this study. The AA + CRE care solution also contained 0.75 g of poloxamer-407 (Wei Ming Pharmaceutical Mfg. Co., Ltd., Taipei, Taiwan) for cleaning, and 0.2 g of ethylenediaminetetraacetic acid (Sigma-Aldrich) for antimicrobial purpose. For pH adjustment, 0.015 g of calcium chloride (Sigma-Aldrich), 0.15 g of potassium chloride (Sigma-Aldrich), 0.45 g of sodium chloride (Sigma-Aldrich), and 0.4 g of sodium phosphate dibasic (Sigma-Aldrich) were added into ddH_2_O in a final volume of 100 mL and a pH of 7.4.

Phosphate-buffered saline (PBS) was obtained from Taiwan Biotech Co., Ltd. (Taoyuan, Taiwan), containing 0.9 g of sodium chloride in 100 mL of ddH_2_O. The artificial tear solution contained lipids, salts, and proteins. The procedure used for the preparation of the lipids + salts solution has been described previously [30], and 2 mg/mL of lysozyme (Sigma-Aldrich) and 0.2 or 2 mg/mL of albumin (Sigma-Aldrich) were added to the lipids + salts solution for further experiments. Normal tear protein contains 2 mg/mL lysozyme and 0.2 mg/mL albumin; however, the albumin concentration of tear protein will increase when ortho-k- lenses are worn, thus 2 mg/mL albumin was also investigated [31,32,33].

### 2.2. In Vitro Tribological Property Analysis for Ortho-K Lenses

**Figure 1:**
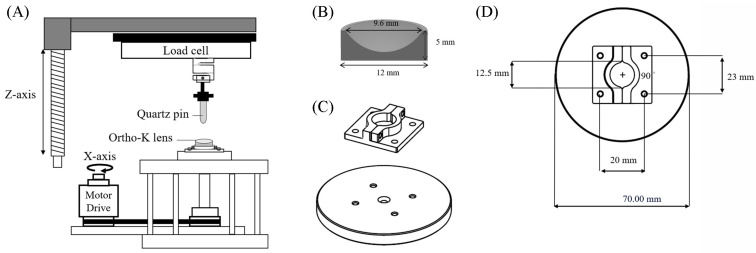
A schematic diagram of an in vitro tribological property analysis for ortho-k lenses. (**A**) The illustration of the platform for tribological testing. (**B**) The cross view of the semi-final product of the ortho-k lens. (**C**) The side view of the specimen holder and the stainless-steel table for the lower stage. (**D**) The top view of the specimen holder on the stainless-steel table.

Tribological analysis was performed with a CETR universal micro-tribometer-2 (UMT-2, Bruker, Campbell, CA, USA). A quartz pin (4 mm diameter) was locked in a holder on the upper stage, which was connected to a sensor, and the sensor was able to sense the loading force and lateral friction force (Figure 1A). The semi-final product of the Boston ES ortho-k lens was placed in the specimen holder, which was made of resin, and fixed by four screws to the lower stainless-steel table which was part of the rotational motor drive (Figure 1B–D). The semi-final ortho-k lens was a cylinder with a curved inner surface (9.6 mm diameter) and its height was 5 mm (Figure 1B). Once the lens was placed onto the lower stage, the upper stage started to move down until the distance between the tip of the quartz pin and the lens was 0.2 mm. The distance between the center of the lens and the quartz pin was 1 mm, and the pin was kept still for 5 min. After adding 100 μL of the tested solution to the curved surface of the lens, the upper stage was then moved downward until the normal load reached 10 mN. The mean pressure of the contact between the eyelid margin and the ocular surface in human subjects has been measured to range from 4.4 to 14.4 mm Hg [34]. The contact area between the quartz pin and the inner surface of the lens was 12.56 mm^2^; thus, the contact pressure was 0.796 kPa, which was around 5.97 mm Hg. Therefore, the normal load of 10 mN was within the normal range of the contact pressure between the eyelid and the ocular surface. The rotation speed was 1 rpm (revolutions per minute), and the rotation time was 900 s. The friction between the ortho-k lens and the cornea only occurs during rapid eye movement, and the duration of rapid eye movement can start for a short amount of time, from 5 to 10 min, and lengthen to 40–60 min [35]. Therefore, the rotation time was set for 900 s. The friction coefficient was friction force divided by normal force, and the friction coefficient from the entire 900 s was averaged. Each liquid was used three times, and an independent semi-final lens was used on each occasion.

### 2.3. Adsorption and Desorption Analyses

The quartz crystal microbalance (QCM, ADS model, ANT Technology Co., Taipei, Taiwan) was used for the adsorption and desorption analyses of different liquids. Approximately 100 μl of 0.5% polymethylmethacrylate solution (PMMA, Sigma-Aldrich) was evenly coated on the surface of the QCM chip using a spin coater (HCS Co., New Taipei City, Taiwan) at 2500 rpm for 15 s. After the PMMA solution was dried, the water contact angle on three random points of the surface was measured using a contact angle meter (Sindatek Instruments Co., Ltd., Taipei, Taiwan) to confirm surface hydrophobicity. The contact angle of the PMMA-coated QCM chip was from 61.48 ± 4.27° to 97.6 ± 1.46°. The thickness of the PMMA-coated film was measured by a surface profiler (Surfcorder ET3000, Kosada Laboratory Co., Ltd., Tokyo, Japan), and the thickness of the PMMA-coated surface was 68 ± 8.5 nm.

**Figure 2:**
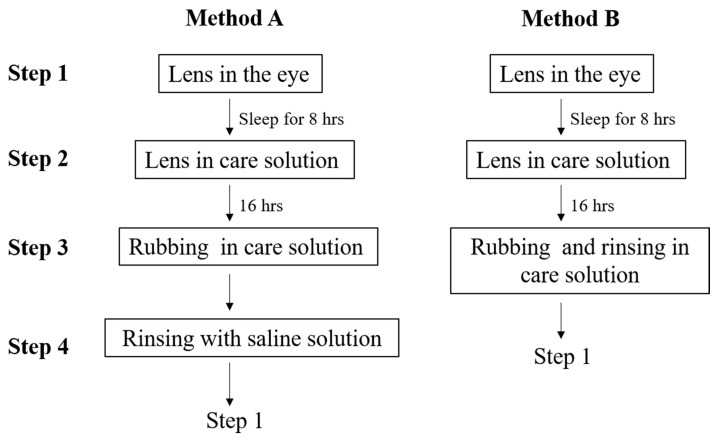
A flowchart for mimicking two different cleaning methods. The only difference is that the lens in method B is not rinsed with saline solution after rubbing and rinsing in care solution, resulting in a lack of Step 4.

To observe the adsorption and desorption of the components in different solutions, two methods of cleaning procedures were investigated. The only difference was whether the lens was rinsed with saline solution before being placed into the eye (Step 4 of method A in Figure 2), and both cleaning methods are recommended by various products. Method B is recommended by MeniCare Plus multipurpose solution, which was used in this study [36], while method A is suggested by other care solutions for gas permeable lens [37]. Therefore, PBS was flowed through the electrode surface of the PMMA-coated chip until the oscillation frequency was maintained within 5 Hz for 10 min. Artificial tear solution was then flowed through the chip surface at a speed of 25 μL per minute (Step 1 in both methods), and the adsorbed components may have caused reduced frequency. When the oscillation frequency was maintained within 5 Hz for 10 min, AA + CRG care solution was then flowed through the PMMA-coated chip (Steps 2 and 3 in both methods) and the changes in oscillation frequency were recorded. For method A cleaning procedure, PBS was flowed through (Step 4 in method A), and a cycle of artificial tear solution and AA + CRG care solution was repeated again. For method B cleaning solution, artificial tear solution was flowed through (back to Step 1 in method B) followed by AA + CRG care solution (Figure 2). PBS was flowed through on the end for both cleaning methods, and each test condition was utilized twice.

### 2.4. Viscosity

The viscosity of four various solutions was measured including artificial tear solution containing 2 mg/mL of lysozyme and 0.2 or 2 mg/mL of albumin, AA + CRG care solution containing 2 mg/mL of lysozyme and 0.2 or 2 mg/mL of albumin. A programmable rheometer (DV-III ultra, Brokfield, Middleboro, MA, USA) with a cone-on-plate fixture in the steady-shear mode was used in this study. A total of 0.5 mL of solution was measured each time, and each solution was tested three times at 25 °C. A rotation of 5 rpm was performed, and viscosity at 19.2 s^−1^ shear rate was recorded. Since the rotation speed in tribological properties analysis and flow speed in adsorption/desorption analyses were both slow, viscosity at a low shear rate should be representative. Each solution was tested three times.

### 2.5. Measurement of Zeta Potential

Four stock solutions were prepared first by dissolving 45 mg of AA, 45 mg of CRG, 100 mg of lysozyme, or 100 mg of albumin in 100 mL of ddH_2_O. A total of 10 mL of a testing solution was then prepared by mixing polysaccharide solution (AA or CRG) with protein solution (lysozyme or albumin) at a 1:1 (*v*/*v*) ratio. The zeta potential of the obtained solution was determined using a Zetasizer (Malvern Panalytical, Malvern, UK). The zeta potential of AA, CRG, albumin, lysozyme, AA + albumin, AA + lysozyme, CRG + albumin, and CRG + lysozyme solution was measured here. Each solution was measured three times.

### 2.6. Turbidity

AA and CRG mixed solutions were prepared at concentrations of 1.25, 2.5, and 5 mg/mL of each polysaccharide. Lysozyme solutions were prepared at concentrations of 1.25, 2.5, and 5 mg/mL. Equal volumes (1 mL each) of AA and CRG mixed solution and lysozyme solution were transferred into the sample chamber, mixed thoroughly by gentle aspiration, and allowed to stand for 30 s. Pictures were taken for each polysaccharides–lysozyme solution, and the transmittance of the mixtures was measured using a UV–Vis spectrophotometer (Metertech SP-8001, Metertech Inc., Taipei, Taiwan) at a wavelength of 600 nm. The 100% transmittance (T%) was corresponded to the absorbance value of ddH_2_O at a wavelength of 600 nm. The turbidity was then calculated as 100 minus the transmittance of the mixed solution, after obtaining the transmittance of each AA + CRG–lysozyme mixed solution. Each solution was analyzed three times.

### 2.7. Statistical Analysis

Differences in the tribological property analysis, viscosity, and turbidity were performed using one-way ANOVA (Analysis Toolpak in Microsoft Excel 2019) followed by Tukey’s post hoc tests for multiple comparisons. A *p*-value of less than 0.05 was considered significant.

## 3. Results

### 3.1. The Effects of Various Solutions on the Friction Coefficient of Ortho-K Lenses

**Figure 3:**
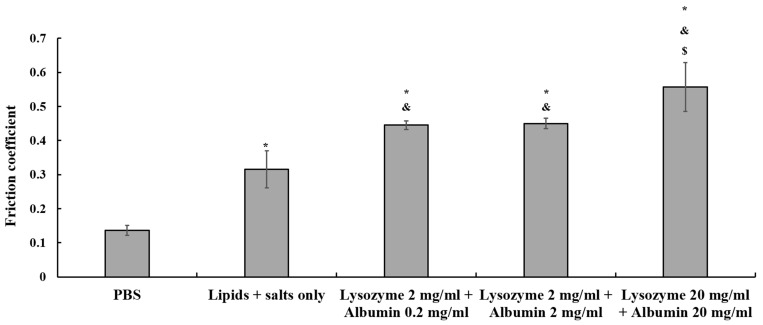
Tribological property analysis when ortho-k lenses are sliding against the quartz pin in different solutions. Friction coefficient is increased when the ortho-k lens is sliding in artificial tear solution containing lysozyme and albumin. * *p* < 0.05 means the friction coefficient of the lens in PBS vs. other conditions. ^&^ *p* < 0.05 means the friction coefficient of the lens in lipids + salts only vs. in different protein concentrations of artificial tear solution. ^$^ *p* < 0.05 means the friction coefficient of the lens in artificial tear solution containing 2 mg/mL of lysozyme and 0.2 mg of albumin vs. in solution containing 2 or 20 mg/mL of lysozyme or albumin. Each condition is analyzed three times, and the bar represents averaged friction coefficient while error bar represents standard deviation.

The friction coefficient was around 0.14 ± 0.01 when the ortho-k lens was sliding against the quartz pin in PBS (Figure 3). In contrast, the friction coefficient increased to 0.32 ± 0.05 when the lens was in the artificial tear solution containing only lipids and salts. Moreover, when 2 mg/mL lysozyme with 0.2 or 2 mg/mL albumin was added to the artificial tear solution, the friction coefficient increased significantly to around 0.45 ± 0.01. The friction coefficient increased to 0.56 ± 0.07 when the lysozyme and albumin concentration was raised up to 20 mg/mL (Figure 3). These results demonstrated that tear film proteins such as lysozyme and albumin would increase friction on the inner surface of the ortho-k lens.

**Figure 4:**
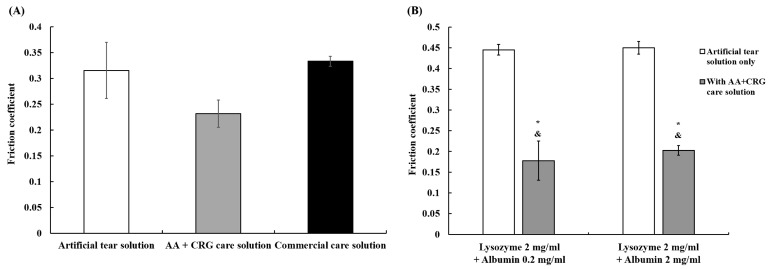
Friction coefficient of various solutions. (**A**) Tribological properties of artificial tear solution (white bar), AA + CRG care solution (gray bar), and commercial care solution (black bar) are similar. (**B**) High friction coefficient caused by proteins is reduced when adding AA + CRG care solution. * *p* < 0.05 or ^&^ *p* < 0.05, friction coefficient of the lens in artificial tear solution containing 2 mg/mL of lysozyme and 0.2 or 2 mg/mL of albumin vs. in artificial tear solution with AA + CRG care solution. Each solution is tested three times, and the bar represents averaged friction coefficient while error bars represent standard deviation.

Compared to the friction coefficient of the artificial tear solution, the AA + CRG care solution significantly reduced the friction to 0.23 ± 0.03 (Figure 4A). The friction coefficient was 0.33 ± 0.01 when the lens was immersed in a commercial care solution for tribological testing, and it was similar to the friction coefficient of the artificial tear solution (Figure 4A). Although AA and CRG showed a lower friction coefficient compared with the artificial tear solution, the decrease was not significant. However, the friction coefficient was reduced significantly when AA + CRG care solution was added to the artificial tear solution regardless of the concentrations of albumin (Figure 4B).

### 3.2. Adsorption and Desorption Behavior of AA + CRG Care Solution on PMMA-Coated Surface

One possibility for lower friction coefficient when the ortho-k lens was sliding in AA + CRG care solution might be that the viscosity of care solution was higher, thus the friction between the lens and the glass could switch from boundary lubrication to mixed lubrication according to the Stribeck curve [38]. The viscosity of various solutions was then investigated, and the result is shown in Table 1. The viscosity was increased significantly when the concentration of albumin increased from 0.2 to 2 mg/mL in the artificial tear solution. The addition of AA and CRG increased the viscosity of the artificial tear solution even more, regardless of the concentrations of proteins.

**Figure 5:**
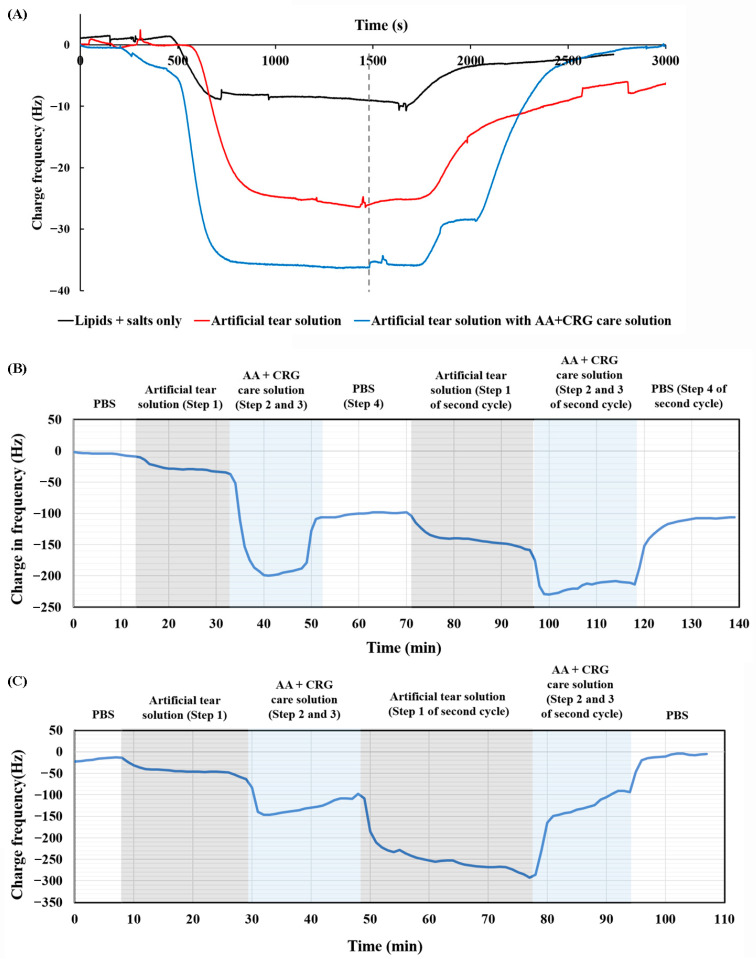
Adsorption and desorption analyses by the QCM system when different solution is flowed through PMMA-coated surface. (**A**) Components are adsorbed when lipid and salt (black line), artificial tear solution (red line), and artificial tear solution with AA and CRG solution (blue line) is flowed through the PMMA-coated surface. Dashed line indicates when PBS is flowed through the chip. (**B**) After two cycles of passing through artificial tear solution (gray area), AA and CRG care solution (blue area), and PBS (white area), frequency is lower than the baseline. Steps are labeled according to cleaning steps in method A of Figure 2. (**C**) After two cycles of passing through artificial tear solution (gray area), AA and CRG care solution (blue area), frequency is back to the baseline when PBS (white area) is flushed on the end of cycle. Steps are labeled according to cleaning steps in method B of Figure 2.

In addition, adsorption and desorption analyses were conducted using the QCM system to ascertain why there was a lower friction coefficient when the ortho-k lens was sliding against the glass in AA + CRG care solution. Some components were adsorbed on the surface of the PMMA-coated chip when the artificial tear solution containing only lipids and salts was flowed through the chip (black line in Figure 5A). The frequency returned close to the original state when PBS was flowed through the chip, demonstrating that the adsorbed components would be taken away easily. In contrast, an artificial tear solution containing lysozyme and albumin was flowed through, resulting in more obvious changes in frequency (red line in Figure 5A). The adsorbed components were not desorbed easily after PBS was flowed through the chip. More components were adsorbed on the PMMA-coated chip when the solution containing AA, CRG, and tear film components was flowed through the chip (blue line in Figure 5A). However, the frequency returned to the original state (0 Hz) when PBS was flowed through, indicating that AA and CRG were able to remove tear film components from the surface of the lens more easily.

The clinical steps of method A (Figure 2) were then investigated, and the results are shown in Figure 5B. Tear film components were not adsorbed to a considerable extent on the PMMA-coated surface during the first cycle (Step 1 in Figure 5B); however, AA and CRG were adsorbed more easily and the frequency was changed to -200 Hz (Steps 2 and 3 in Figure 5B). When PBS was flowed through the chip, the frequency only returned to −100 Hz (Step 4 in Figure 5B). During the second cycle, more components were adsorbed on the PMMA-coated surface after the artificial tear solution and AA + CRG care solution were subsequently flowed through and the frequency was changed to around −220 Hz (Steps 2 and 3 in the second cycle of Figure 5B). Interestingly, the frequency returned to −100 Hz once again when PBS flowed through the surface (Step 4 in the second cycle of Figure 5B), demonstrating that AA and CRG could remove tear film components more easily.

The result of cleaning method B (Figure 2) is shown in Figure 5C. Similarly to method A, AA and CRG were adsorbed more easily on the PMMA-coated surface (Step 2 and 3 in Figure 5C) compared with tear film components (Step 1 in Figure 5C). However, AA and CRG also flowed away from the surface quickly because the frequency was changed from −150 to −100 Hz within 20 min at the first cycle. In the beginning of the second cycle, tear film components were adsorbed on the surface much more easily than the first cycle (Step 1 in the second cycle of Figure 5C) and the frequency even reached −300 Hz. Interestingly, AA and CRG this time could bring away tear film components and the frequency was returned to −100 Hz (Steps 2 and 3 in the second cycle of Figure 5C). The frequency could even be back to the initial baseline on the end after the chip being rinsed, with PBS suggesting that AA and CRG could bring tear film components away from the PMMA-coated surface efficiently.

### 3.3. The Potential Interaction Between AA + CRG Care Solution and Tear Film Proteins

**Figure 6:**
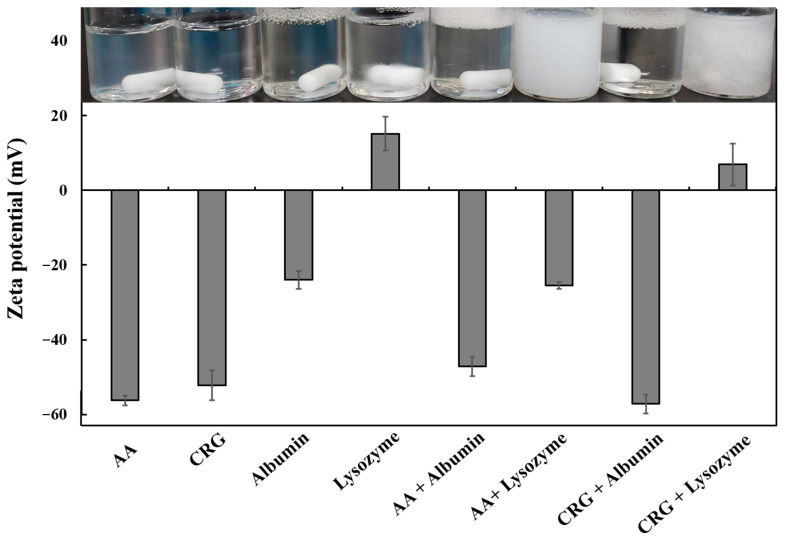
The zeta potential and representative photos of various solutions. Gray bars represent averaged zeta potential from three independent tests, and error bars represent standard deviation.

In order to understand how AA and CRG could bring away adsorbed tear film components, the interaction between AA/CRG and lysozyme/albumin was first investigated. One of the potential interactions was electrostatic forces. By analyzing the zeta potential of AA, CRG, lysozyme, or albumin alone, only lysozyme presented as positively charged while the other three molecules were negatively charged (Figure 6). The zeta potential result still showed the mixed solution was negatively charged and the solution was transparent when AA or CRG was mixed with albumin. In contrast, the mixed solution was not transparent when AA or CRG was mixed with lysozyme. The zeta potential of the AA + lysozyme solution was less negatively charged (from −56.2 ± 1.3 to −25.5 ± 1.0 mV), and the zeta potential of the CRG + lysozyme solution was even changed to positively charged when compared with AA or CRG alone (Figure 6). These results demonstrated that there should be electrostatic attraction between AA/CRG and lysozyme.

**Figure 7:**
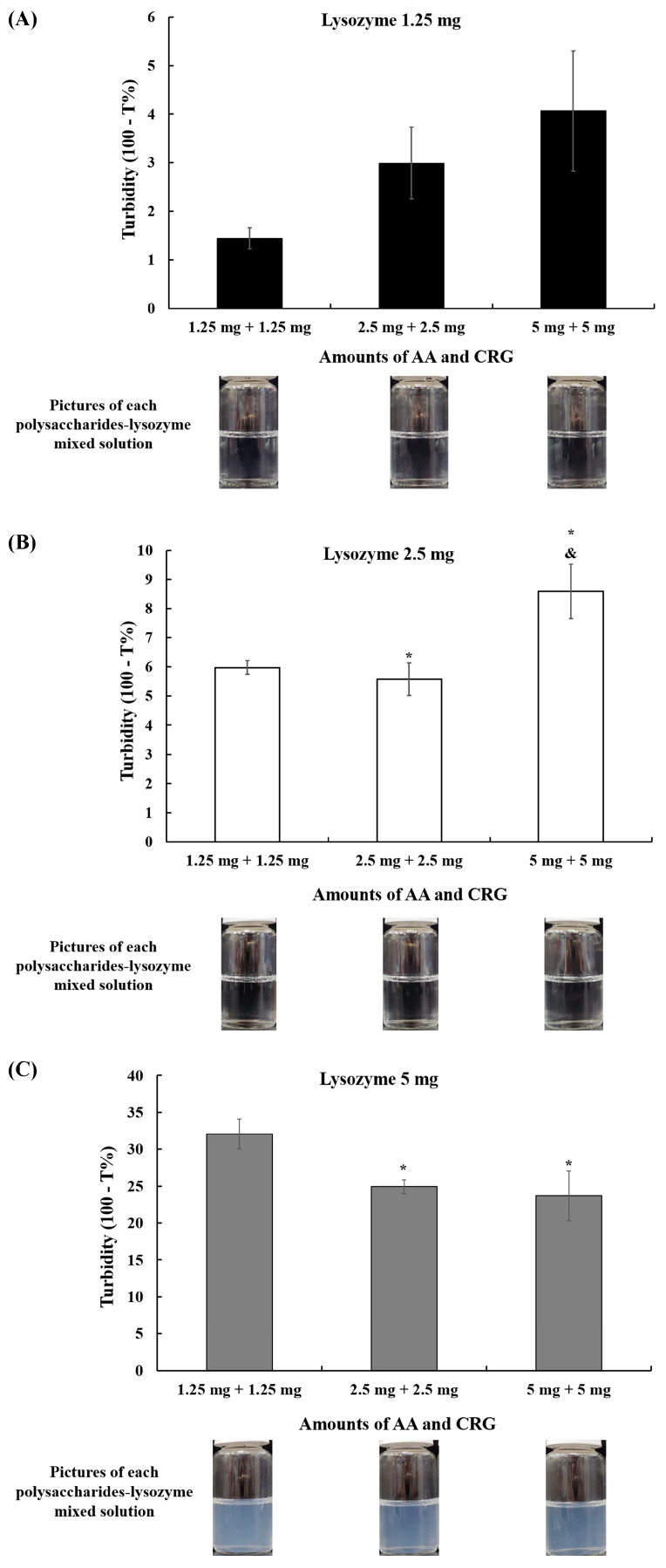
The turbidity and the representative photos of various lysozyme–AA + CRG mixed solutions when the amounts of lysozyme are 1.25 (**A**), 2.5 (**B**), or 5 mg (**C**) in the solution. Black, white, or gray bars represent averaged turbidity from three measurements, and error bars represent standard deviation. * *p* < 0.05 or ^&^ *p* < 0.05 indicates a significant difference compared with the mixed solution containing 1.25 mg or 2.5 mg of AA and CRG, respectively.

The mixed solution of AA + lysozyme or CRG + lysozyme was turbid, and some suspension or participates were observed in the CRG + lysozyme solution, suggesting an electrostatic attraction might lead to the formation of a lysozyme–polysaccharides complex. In order to understand when these complexes might be occurring, the turbidity of various amounts of AA, CRG, and lysozyme mixtures was investigated. The solution was transparent and the turbidity was under 9% regardless of the amounts of AA and CRG when 1.25 or 2.5 mg of lysozyme were in the mixture (Figure 7A,B). In contrast, the turbidity was higher than 23% when 5 mg of lysozyme was in the mixture and a turbid solution was then observed (Figure 7C). Interestingly, the turbidity increased when the amounts of AA and CRG were rising in the solution containing 1.25 mg or 2.5 mg of lysozyme. However, the turbidity was decreased significantly when the amounts of AA and CRG were increasing in the mixed solution containing 5 mg of lysozyme. These results suggested that the lysozyme–polysaccharides complex might mainly depend on the amounts of lysozyme rather than the amounts of AA and CRG.

## 4. Discussion

The in vitro tribological property analysis demonstrated that tear film proteins increase the friction coefficient of ortho-k lenses, and AA + CRG care solution could significantly reduce the high friction coefficient in this study (Figure 3 and Figure 4). Previous studies have demonstrated that the interaction between the eyelid, cornea, and contact lens falls into boundary lubrication according to the Stribeck curve [13,39,40]. Since the contact pressure and rotation speed were not changed, a reduction in the friction coefficient in artificial tear solution containing AA + CRG care solution could likely be influenced by viscosity and changes in surface interactions. Indeed, viscosity was increased significantly when AA + CRG care solution was added into artificial tear solution regardless of concentrations of tear film proteins (Table 1). Clinically, AA + CRG care solution cannot be dripped into the eye directly because it is not made for eyedrops. In addition, the viscosity of each solution was measured at its static state and it might not reflect the frictional behavior of AA + CRG care solution in dynamic conditions such as rapid eye movement. Therefore, it was more critical to investigate whether AA + CRG care solution can bring away adsorbed tear film components from the lens surface.

The QCM analysis demonstrated that the adsorption of tear film lipids on the PMMA surface was weak, resulting in them being washed away easily by PBS (black line in Figure 5A). When proteins were added to the artificial tear solution, they were adsorbed on the PMMA-coated surface and not easily washed away by PBS. The above results show that a greater number of components were adsorbed on the surface when a mixture of protein-containing artificial tear solution and AA + CRG care solution was flowed through the surface (red line in Figure 5A). However, the rate of adsorption was not strong, resulting in them being easily washed away by PBS (blue line in Figure 5A). Interestingly, some portion of the adsorbed components would still be kept on the PMMA-coated surface if PBS was passed immediately after the AA + CRG care solution (Steps 2 and 3 in the second cycle of Figure 5B). In contrast, adsorbed components could be completely removed from the surface if only the AA + CRG care solution was applied for rinsing the lens (Figure 5C and Step 4 in cleaning method B in Figure 2). The adsorption and desorption results suggest that AA + CRG care solution should be used for removing adsorbed proteins and should not be washed by PBS before the ortho-k lens is placed into the eye by applying cleaning method B.

The possible mechanism of how AA and CRG could bring away adsorbed tear film components, mainly proteins, from the surface was then investigated. The interaction between polysaccharides and proteins has been shown to be divided into electrostatic interactions, hydrophobic interactions, and hydrogen bonding [41]. The isoelectric point of lysozyme and albumin is 11.4 and 5.16, respectively, and AA and CRG are negatively charged when the pH value of the artificial tear solution is neutral [42,43]. Indeed, zeta potential analysis demonstrated the above results (Figure 6). Although AA and CRG could interact with lysozyme via electric attraction, the same attraction does not occur between AA/CRG and albumin (Figure 6). Another possible mechanism behind the removal of adsorbed proteins away from the PMMA-coated surface is that AA/CRG and lysozyme/albumin form biopolymers via hydrophobic–hydrophobic interaction [44]. The formation of biopolymers of polysaccharides and proteins might result in the exposure of hydrophilic sites on the surface and then be easily rinsed away by PBS or AA + CRG care solution (Figure 5B,C). The turbidity results suggested that AA + CRG and lysozyme might form aggregates or complexes because the transmittance was decreased and the turbid solution was obvious when the amounts of lysozyme were increased (Figure 7). However, whether AA + CRG and lysozyme complexes can form hydrophobic–hydrophobic bonding will require further investigation such as Fourier Transform Infrared Spectrometry analysis. In addition, whether similar phenomenon could occur between AA + CRG and albumin will also need further analysis.

**Figure 8:**
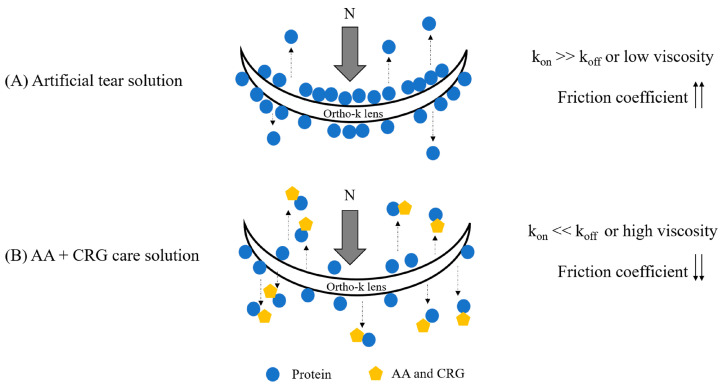
The potential mechanism of how AA and CRG (yellow pentagons) can bring away adsorbed proteins (blue circles) from the ortho-k lens and cause lower friction coefficient. (**A**) When the lens is immersed in artificial tear solution, the rate of protein adsorption is faster than the rate of desorption (k_on_ >> k_off_) or low viscosity of artificial tear solution itself resulting in higher friction coefficient. (**B**) Electrostatic attraction and biopolymers between AA/CRG and proteins may form to bring away adsorbed protein or high viscosity of a mixed solution, resulting in lower friction coefficient of the ortho-k lens. The dashed black arrows indicate desorption of proteins or polysaccharide–protein complexes. The normal load (gray arrow, represented by N) of frictional tests is set at 10 mN, and black arrows indicate the changes in friction coefficient.

A previous study has shown that the friction coefficient of soft contact lenses is increased when the concentration of lysozyme is increased [45]. The authors proposed that the protein–protein interaction increases under a high concentration of lysozyme, resulting in more adsorbed protein, less protein structural changes, and a higher friction coefficient [45]. The friction coefficient of the inner surface of the ortho-k lens was also increased when proteins were added into the artificial tear solution or when the concentration of proteins was increased (Figure 3). Therefore, a potential mechanism of how AA and CRG provided lubrication is proposed in Figure 8. More proteins are adsorbed on the lens, resulting in a stronger protein–protein interaction (k_on_ >> k_off_) and an increase in the friction coefficient, when the ortho-k lens is sliding against a quartz glass in an artificial tear solution with higher concentrations of proteins (Figure 8A). While AA + CRG care solution was added into an artificial tear solution or passed through a PMMA-coated surface, AA + CRG could bring away proteins from the surface possibly via electrostatic or hydrophobic–hydrophobic interactions resulting in a weaker protein–protein interaction (k_on_ << k_off_) and a reduction in the friction coefficient (Figure 8B). Additionally, a higher viscosity of mixed AA + CRG care solution and tear film proteins may suggest that if some AA and CRG remain on the lens surface when cleaning method B was applied, the ortho-k lens friction coefficient can also be decreased simply by switching from boundary lubrication to mixed lubrication. AA and CRG may also be incorporated into the surface coating materials of ortho-k lenses, and being coated on the lens surface may also be effective for lowering the friction coefficient.

Although the in vitro tribological property analysis for ortho-k lenses established here could distinguish the frictional behavior of various solutions, using a quartz glass pin as the counter surface could not reflect human cornea tissue. The hardness of quartz glass is high, indentation values can be around 9–15 GPa [46,47], thus the contact mechanics are dominated by the compliance of PMMA. Quartz is hydrophilic but chemically stable and can be reused reproducibly, which reduces the variability from changing counter surface chemistry. The friction changes can then come from the ortho-k lenses and tested solutions, not from contamination or adsorption on the counter surface. However, using a quartz glass pin becomes one of the limitations in this study. In the future, biomaterials whose hardness are close to a human cornea should be applied as the counter surface to reflect actual in vivo frictional behavior of ortho-k lenses. Additionally, further investigation of the tribological performance of other commercial care solutions is necessary to substantiate whether the AA + CRG formulation provides superior lubricating efficacy. In this study, only lysozyme and albumin were used to investigate how the frictional property, viscosity, and adsorption/ desorption behavior of AA and CRG on ortho-k lens surfaces are influenced. Tear film proteins also contain mucin, lactoferrin, lipocalin, secretory IgA, etc. [48]. Whether these proteins contribute to an even higher friction coefficient or provide better lubrication will require further investigation. In addition, only lambda-carrageenan was mixed with AA in the current study. Since iota- or kappa-carrageenan has been shown to be a good lubricating material under certain conditions, these two carrageenans should be formulated with AA to investigate whether they can provide better lubrication than lambda-carrageenan. However, the current results have provided a solution for ortho-k lens wearers to reduce the friction coefficient on the inner surface of the lens, leading to a reduction in the number of clinical complications such as discomfort.

## 5. Conclusions

The present study demonstrates that alginate acid (AA) and lambda-carrageenan (CRG) care solution can effectively reduce the elevated friction coefficient on the inner surface of the ortho-k lens caused by tear film proteins using an in vitro tribological property analysis for ortho-k lenses that was established here. The frictional improvement was accompanied by an increase in solution viscosity and altered adsorption–desorption behavior, indicating that both fluid properties and surface interactions contribute to the observed lubrication effects. QCM analysis further revealed that AA + CRG promotes the removal of protein deposits from PMMA-coated surfaces under specific cleaning conditions. These findings suggest that the underlying mechanisms may involve electrostatic interactions between negatively charged polysaccharides and positively charged proteins, as well as the formation of polysaccharide–protein complexes that reduce protein–protein association on the lens surface. The results offer new insights into how AA + CRG-based formulations can modulate tribological behavior in protein-rich tear environments and point to promising strategies for improving the comfort and safety of ortho-k lens wear, thus providing the development of next-generation lens care solutions and surface modification technologies.

## Figures and Tables

**Table 1:** The viscosity of various solutions and the results represented as mean ± standard deviation. * *p* < 0.05, viscosity of various solutions vs. viscosity of artificial tear solution containing 2 mg/mL lysozyme and 0.2 mg/mL albumin. ^&^ *p* < 0.05 when comparing viscosity with artificial tear solution containing 2 mg/mL of lysozyme and 2 mg/mL of albumin. Each solution is measured three times.

Group	Artificial Tear Solution Containing 2 mg/mL Lysozyme and 0.2 mg/mL Albumin	Artificial Tear Solution Containing 2 mg/mL Lysozyme and 2 mg/mL Albumin	AA + CRG Care Solution Containing 2 mg of Lysozyme and 0.2 mg/mL of Albumin	AA + CRG Care Solution Containing 2 mg/mL of Lysozyme and 2 mg/mL of Albumin
Viscosity (mPa·s) at 19.2 s^−1^	1.15 ± 0.16	1.65 ± 0.17 *	12.23 ± 0.02 *^,&^	11.92± 0.05 *^,&^

## Data Availability

Data is contained within the article.
